# Supplementary material for: Bimetallic nanoparticles and biochar produced by Adansonia Digitata shell and their effect against tomato pathogenic fungi
Source: PeerJ. 2024 Mar 1;12:e17023. doi: 10.7717/peerj.17023 (PMC10911075; doi:10.7717/peerj.17023)
Supplement: Supplemental Information 4 [file peerj-12-17023-s004.docx]

Figure S1: Plates indicating the fungal growth in media treated with AgNPs, FeONPs , Bimetallic NPs and biochar in relation to control:


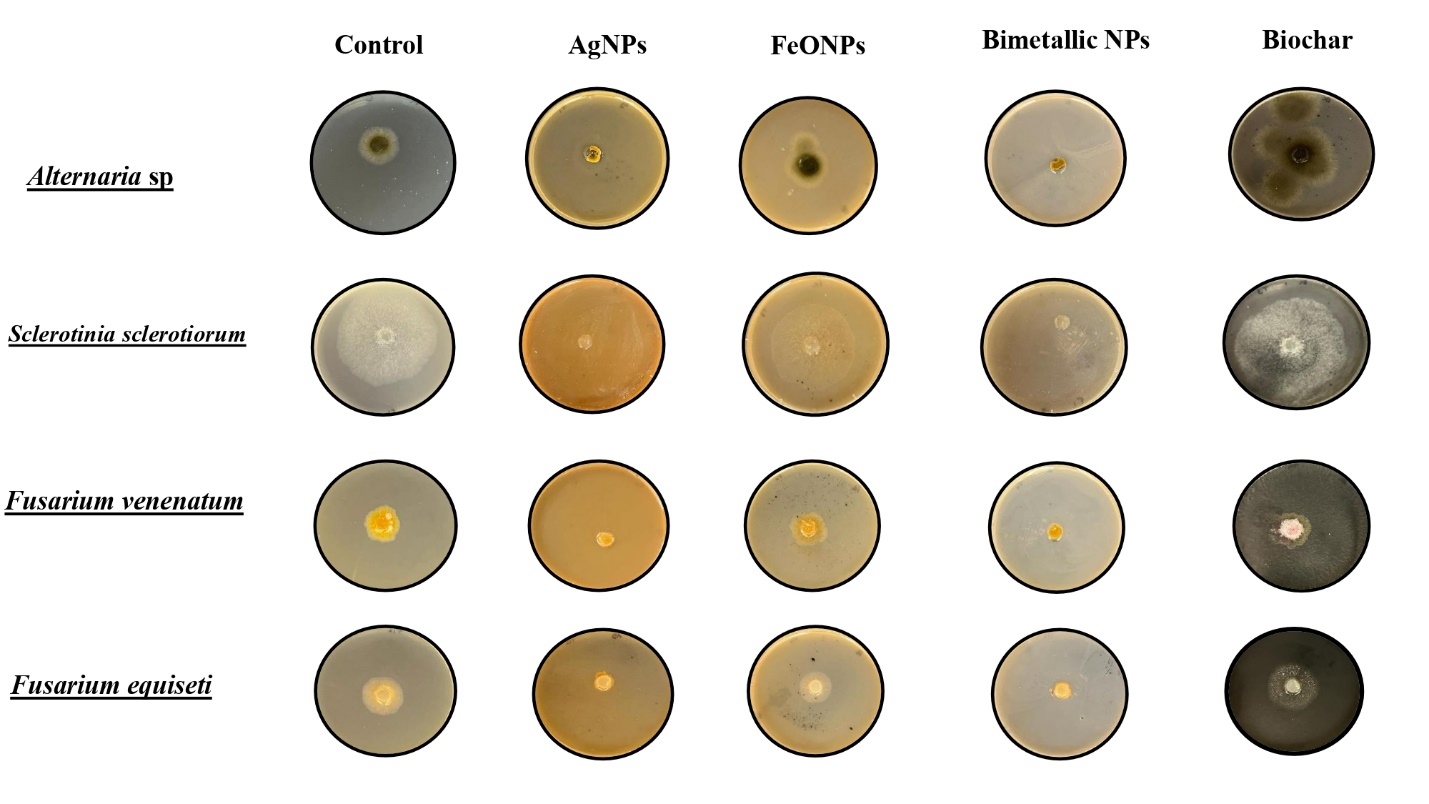


Figures S2: The FTIR spectra of *A. digitata* fruit shell extract, phyto-fabricated nanoparticles, and biochar.

1.
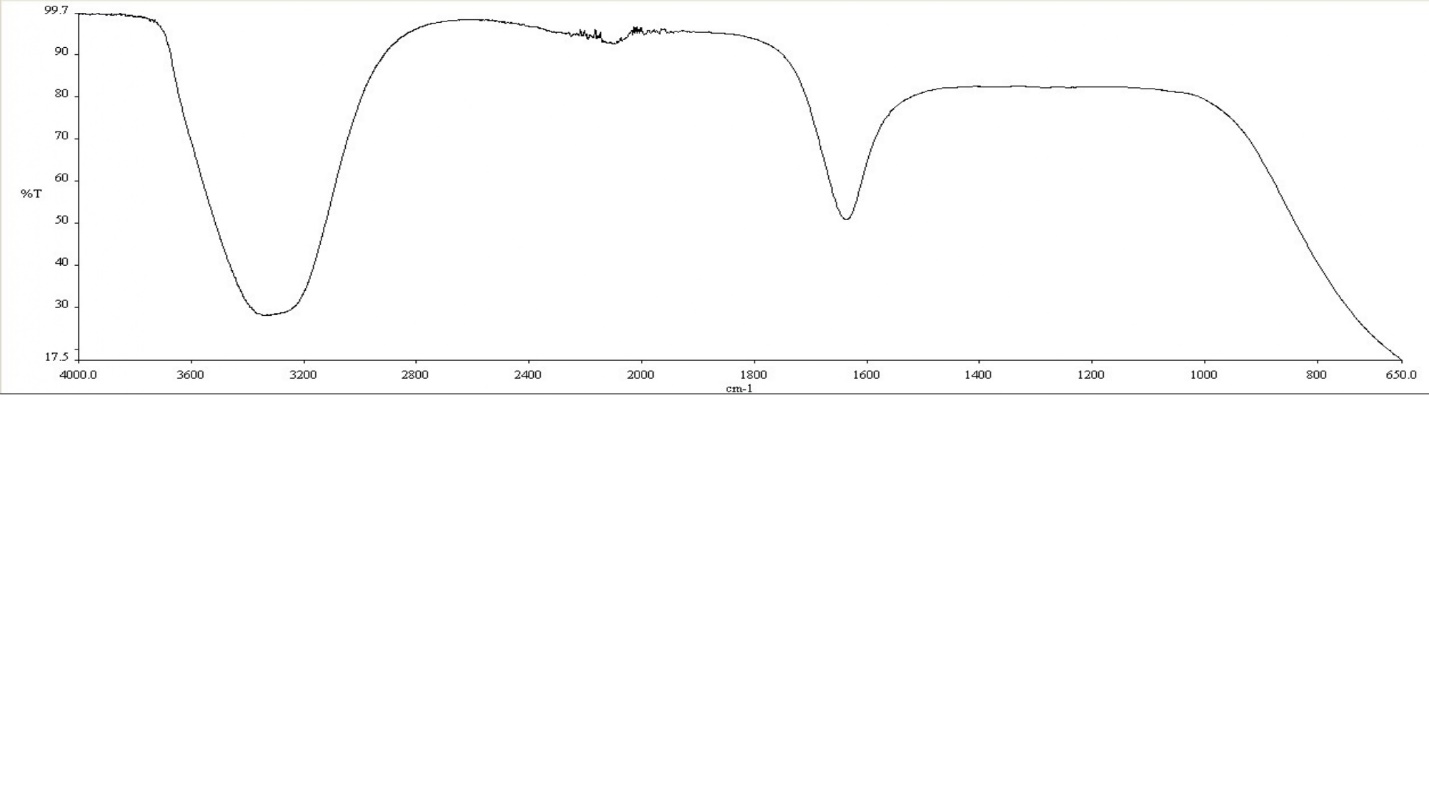
**The FTIR spectral analysis of plant extract:**
2.
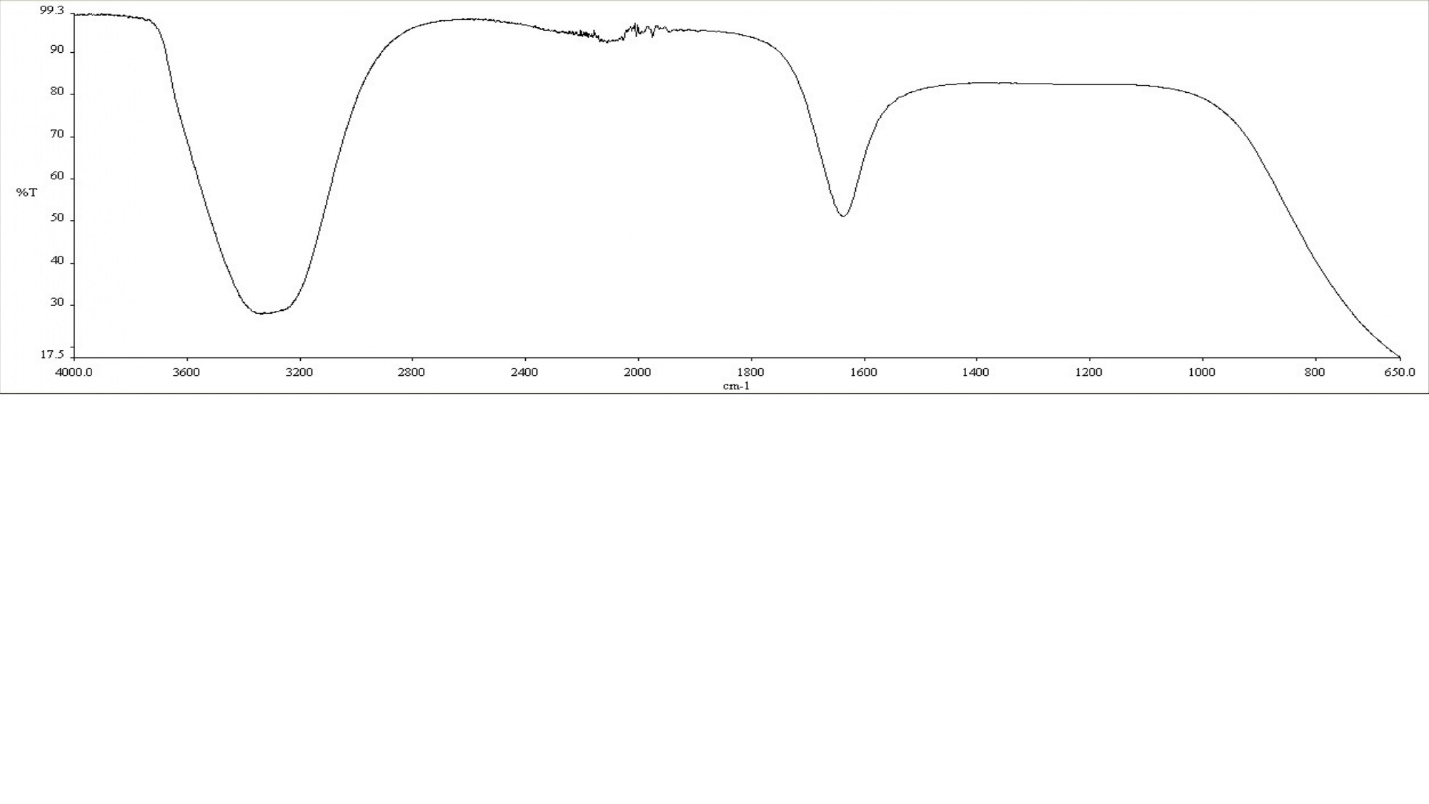
 **The FTIR spectral analysis of AgNPs:**
3.
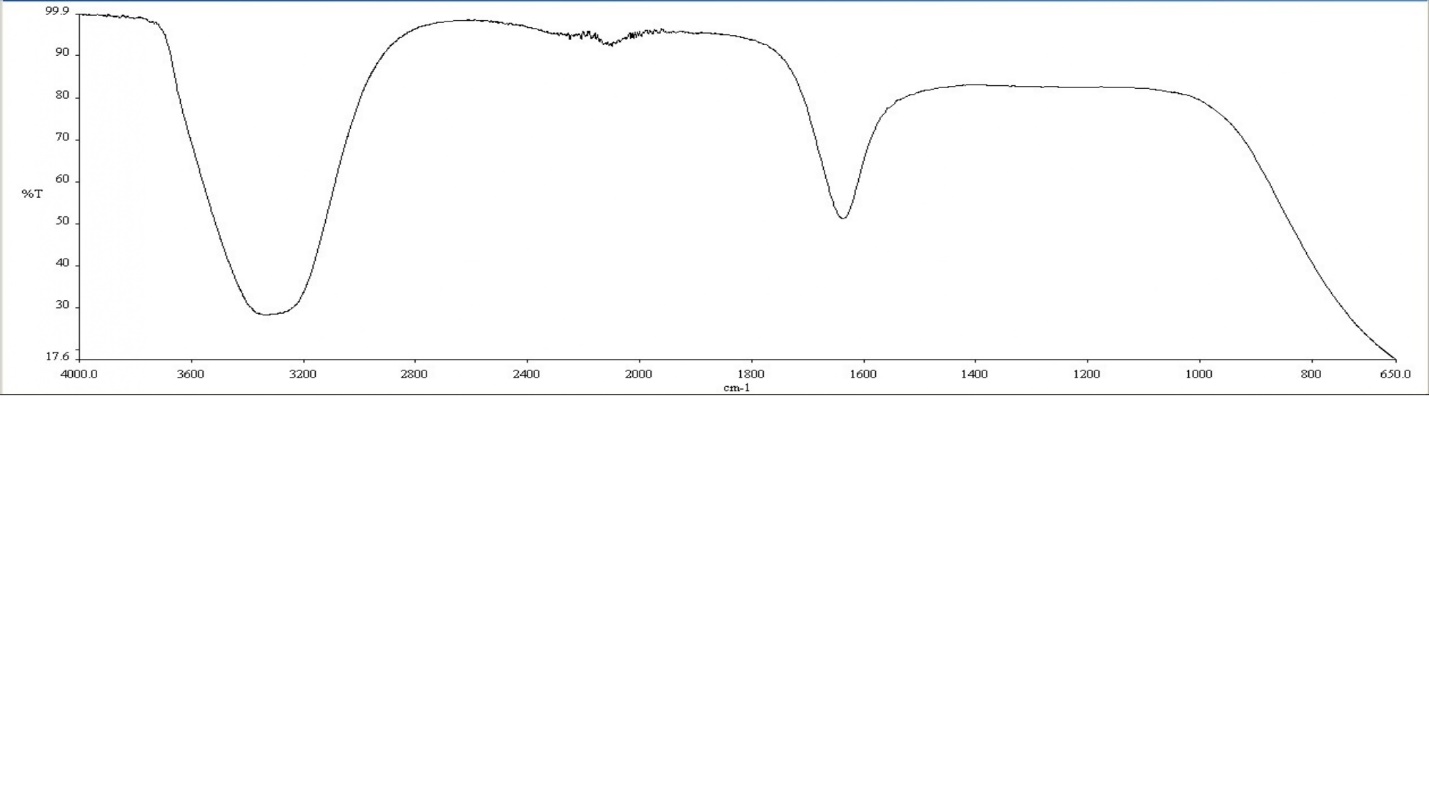
**The FTIR spectral analysis of FeONPs:**
4. **The FTIR spectral analysis of bimetallic NPs:**


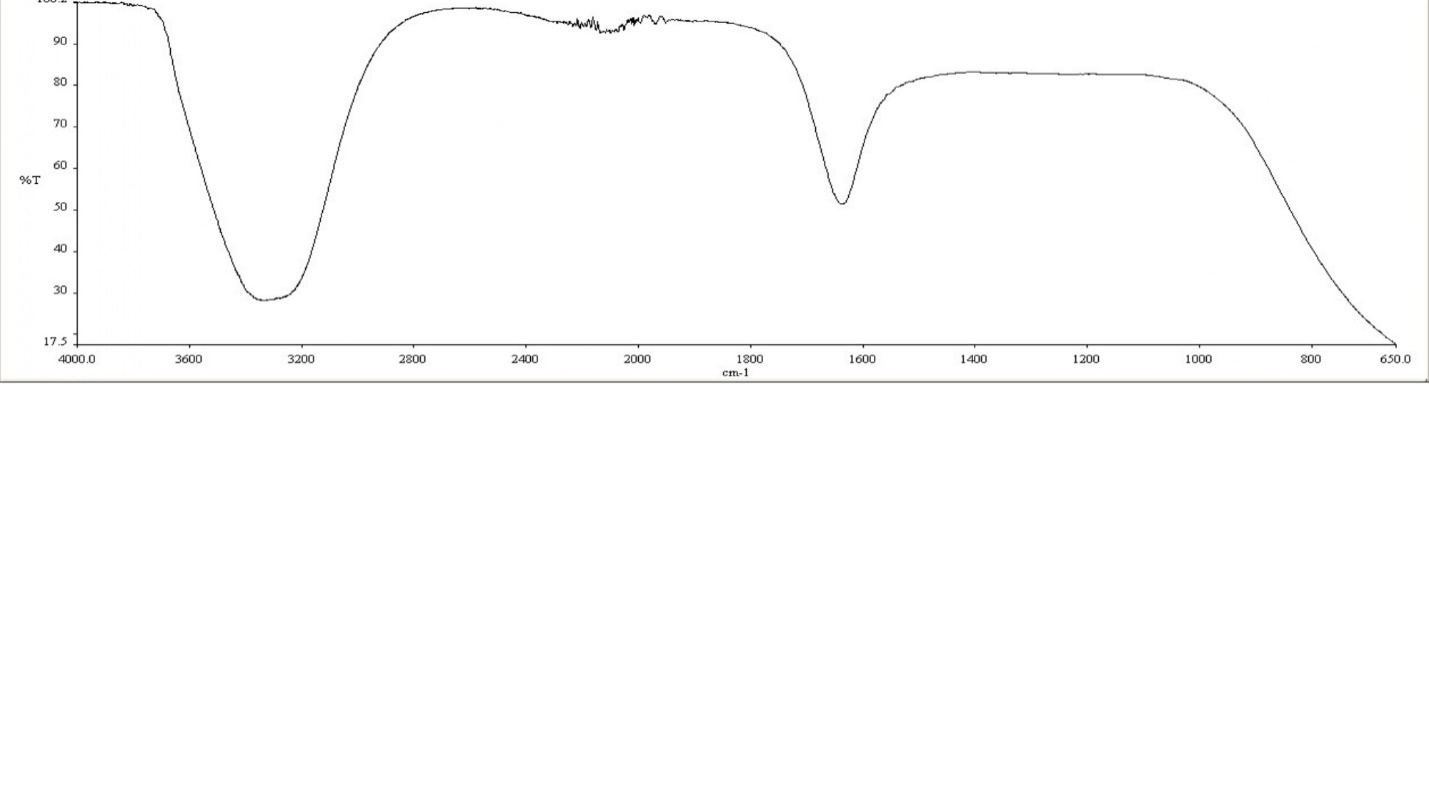


1. **The FTIR spectral analysis of Biochar:**


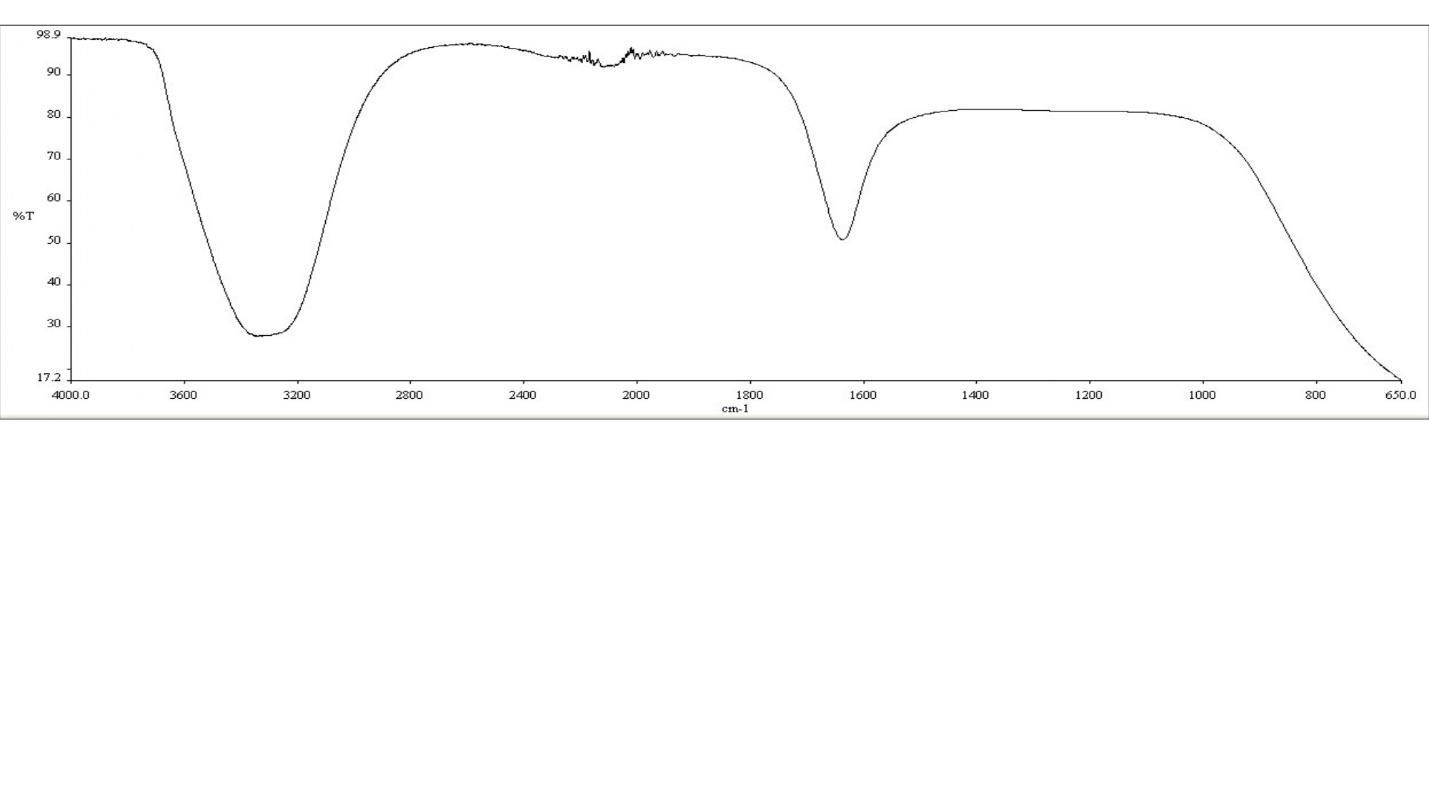


**Table S1:**

**The major three peaks from the FTIR analysis:**

| Materials | Peak 1 | Peak 2 | Peak 3 |
| --- | --- | --- | --- |
| Plant extract | 3336 | 2023 | 1635 |
| AgNPs | 3337 | 2023 | 1636 |
| FeONPs | 3337 | 2020 | 1637 |
| Bimetallic | 3337 | 2023 | 1637 |
| Biochar | 3341 | 2008 | 1636 |
